# Treatment with a novel oleic-acid–dihydroxyamphetamine conjugation ameliorates non-alcoholic fatty liver disease in obese Zucker rats

**DOI:** 10.1242/dmm.019919

**Published:** 2015-10-01

**Authors:** Juan M. Decara, Francisco Javier Pavón, Juan Suárez, Miguel Romero-Cuevas, Elena Baixeras, Mariam Vázquez, Patricia Rivera, Ana L. Gavito, Bruno Almeida, Jesús Joglar, Rafael de la Torre, Fernando Rodríguez de Fonseca, Antonia Serrano

**Affiliations:** 1 Unidad Gestión Clínica de Salud Mental, Instituto de Investigación Biomédica de Málaga (IBIMA), Hospital Regional Universitario de Málaga/Universidad de Málaga, Málaga, 29010 Spain; 2 CIBER de Fisiopatología de la Obesidad y la Nutrición (CIBERobn), Instituto de Salud Carlos III (ISCIII), Madrid, 28029 Spain; 3 Institut Hospital del Mar d'Investigacions Mèdiques (IMIM), Neurosciences Program, Barcelona, 08003 Spain; 4 Facultat de Ciencies de la Salut i de la Vida, Universitat Pompeu Fabra (CEXS-UPF), Barcelona, 08002 Spain; 5 Departamento de Química Biológica y Modelización Molecular, Instituto de Química Avanzada de Cataluña (IQAC-CSIC), Barcelona, 08034 Spain

**Keywords:** Obesity, Non-alcoholic fatty liver disease, Zucker rats, Cannabinoid type 1 receptor, CB_1_, Peroxisome proliferator activated-receptor α, PPAR-α

## Abstract

Fatty liver disease is one of the main hepatic complications associated with obesity. To date, there are no effective treatments for this pathology apart from the use of classical fibrates. In this study, we have characterized the *in vivo* effects of a novel conjugation of oleic acid with an amphetamine derivative (OLHHA) in an animal model of genetic obesity. Lean and obese Zucker rats received a daily intraperitoneal administration of OLHHA (5 mg kg^−1^) for 15 days. Plasma and liver samples were collected for the biochemical and molecular biological analyses, including both immunohistochemical and histological studies. The expression of key enzymes and proteins that are involved in lipid metabolism and energy homeostasis was evaluated in the liver samples. The potential of OLHHA to produce adverse drug reactions or toxicity was also evaluated through the monitoring of interactions with hERG channel and liver cytochrome. We found that OLHHA is a drug with a safe pharmacological profile. Treatment for 15 days with OLHHA reduced the liver fat content and plasma triglyceride levels, and this was accompanied by a general improvement in the profile of plasma parameters related to liver damage in the obese rats. A decrease in fat accumulation in the liver was confirmed using histological staining. Additionally, OLHHA was observed to exert anti-apoptotic effects. This hepatoprotective activity in obese rats was associated with an increase in the mRNA and protein expression of the cannabinoid type 1 receptor and a decrease in the expression of the lipogenic enzymes FAS and HMGCR primarily. However, changes in the mRNA expression of certain proteins were not associated with changes in the protein expression (i.e. L-FABP and INSIG2). The present results demonstrate that OLHHA is a potential anti-steatotic drug that ameliorates the obesity-associated fatty liver and suggest the potential use of this new drug for the treatment of non-alcoholic fatty liver disease.

## Abstract (precis)

**Drug Discovery Collection:** OLHHA is a safe drug with hepatoprotective and anti-steatotic effects on non-alcoholic fatty liver disease in obese rats.

## INTRODUCTION

Both alcoholic fatty liver (AFL) and non-alcoholic fatty liver disease (NAFLD) are the result of an excessive fat accumulation in the liver. Whereas AFL is associated with a long-term intake and abuse of alcohol, the development of NAFLD is produced in the absence of a history of excessive alcohol consumption. Nonetheless, both AFL and NAFLD can progress to more severe liver diseases, including steatohepatitis, fibrosis or cirrhosis.

NAFLD has consistently shown a dramatic increase in prevalence over the last few decades in Western countries, reaching the 20-40% prevalence rate and becoming an important health issue (Farrell and Larter, 2006). Several studies have reported that NAFLD is closely related to the metabolic syndrome (Bugianesi et al*.*, 2005; Chavez-Tapia et al*.*, 2006) and is a risk factor associated with obesity, type II diabetes mellitus, insulin resistance, hypertension and hypertriglyceridemia (Marchesini et al., 2003). Given that the pathogenesis of NAFLD is not yet fully elucidated, animal models can provide an important complement to a better understanding of this disease. As a result of the strong association between obesity and NAFLD, animal models of obesity are suitable to study the course and evolution of NAFLD. For instance, the obese Zucker rat is a model of genetic obesity caused by a spontaneous mutation in the leptin receptor that displays hyperphagia, hyperinsulinemia, hypertriglyceridemia and development of fatty liver in adulthood.

Although there are no pharmacological therapies for reversing and preventing NAFLD, several synthetic and natural fatty acid-derived molecules that have been identified as potential anti-obesity drugs may represent an interesting pharmacological tool. These lipid compounds act basically through two types of receptors related to appetite regulation and/or energy homeostasis, the cannabinoid type 1 receptor (CB_1_) and the peroxisome proliferator activated-receptor α (PPAR-α).

Numerous studies suggest the involvement of CB_1_ activation in the development of fatty liver associated with obesity. For instance, a study using an *in vitro* model of steatosis has demonstrated a downregulation of CB_1_ (De Gottardi et al., 2010). Other studies in the liver have reported that the activation of CB_1_ increases the expression of lipogenic genes and inhibits fatty acid oxidation, whereas pretreatment with a CB_1_ antagonist prevents this effect (Jourdan et al., 2010; Osei-Hyiaman et al., 2005, 2008). In fact, CB_1_ antagonists have shown hepatoprotective activity, and treatment with rimonabant reverses and prevents the fatty liver in obese Zucker rats (Gary-Bobo et al., 2007) and reduces the liver fat in abdominally obese subjects (Despres et al., 2009).

**TRANSLATIONAL IMPACT**

**Clinical issue**

Non-alcoholic fatty liver disease (NAFLD) is a common disorder that is caused by excessive fat accumulation in the liver in the absence of a history of alcohol abuse. Although it has been considered as a benign condition for a long time, it can progress to a more severe non-alcoholic steatohepatitis or cirrhosis. Currently, there are no safe and effective pharmacological therapies for NAFLD, and major efforts are being dedicated to identification of new targets and strategies for reversing and preventing NAFLD. Given that NAFLD is one of the consequences associated with obesity, new anti-obesity drugs might represent an interesting pharmacological tool. Substantial evidence suggests that the blockade of the cannabinoid type 1 receptor (CB_1_) and/or the activation of the peroxisome proliferator activated-receptor α (PPAR-α) have anti-obesity effects and, therefore, might represent a feasible approach for NAFLD treatment.

**Results**

In this study, the authors have shown that a novel conjugation of oleic acid with an amphetamine derivative (OLHHA) is effective for the treatment of NAFLD. This compound has been previously reported to have affinity for both the CB_1_ and PPAR-α and it displays anti-obesity properties. Chronic administration of OLHHA to obese Zucker rats reduced both hepatic lipid accumulation and circulating triglyceride levels. These animals also displayed a general improvement in the profile of plasma parameters related to liver damage. This effect was associated with an anti-apoptotic activity and with a downregulation of the expression of enzymes involved in the biosynthesis of lipids in the liver, thus reducing lipid production. In addition, toxicity studies revealed that OLHHA is a drug with a safe pharmacological profile.

**Implications and future directions**

This study provides evidence that this novel anti-obesity drug has a hepatoprotective effect and potent antioxidant properties, suggesting its potential therapeutic application for the treatment of NAFLD. Although OLHHA has been reported to have affinity for both the CB_1_ and the PPAR-α, further studies are needed to establish the specific mechanism of action of this drug. The safe pharmacological profile on the heart human ether-à-go-go-related gene (hERG) channel (which is sensitive to drug binding and can trigger a drug-induced long QT syndrome) and liver cytochromes suggests this drug as a suitable candidate to progress towards clinical trials for NAFLD.

PPAR-α is involved in the control of multiple aspects of energy balance, playing a pivotal role in the control of the transcription of genes involved in lipid metabolism (Rosen, 2003). Among the several endogenous ligands proposed for PPAR-α, *N*-oleoylethanolamine (OEA) acts as a lipid mediator of satiety regulated by feeding (Rodríguez de Fonseca et al., 2001). Besides inducing satiety, OEA is also involved in the control of energy expenditure and fat utilization. In the liver, the PPARα-mediated effects of OEA have been thoroughly investigated (Thabuis et al., 2008). Previous studies have demonstrated that OEA is able to prevent liver damage, reducing the hepatic lipid content (Fu et al., 2005; Guzman et al., 2004; Serrano et al., 2008b).

In this context, it has been described that a combinational therapy with CB_1_ blockers and PPAR-α activators improves the individual effect of each drug in obese Zucker rats, resulting in a decrease of food intake, body weight gain and liver fat depots and an improvement of liver function (Pavón et al*.*, 2008; Serrano et al*.*, 2008a). Consequently, the development of new drugs with dual activity as ligands for CB_1_ and PPAR-α has been reported in recent years (Alvarado et al*.*, 2008; Pérez-Fernández et al., 2011). Recently, we have synthesized and characterized a series of compounds derived from fatty acids conjugated with 3,4-methylenedioxymethamphetamine metabolites as anti-obesity drugs (Almeida et al., 2010). Among these novel molecules, the *N*-[1-(3,4-dihydroxyphenyl)propan-2-yl]oleamide (OLHHA) has inhibitory effects on feeding behaviour and produces antioxidant effects, acting as a potent inhibitor of low-density lipoprotein oxidation, as disclosed in the International Patent Application No. WO 2011076966 A1 (De la Torre et al., 2011). Given that OLHHA has been reported to have affinity for both the CB_1_ and PPAR-α in the nanomolar range, these described effects may be mediated by an interaction between CB_1_ and PPAR-α.

Considering that OLHHA represents a new pharmacological approach to the treatment of obesity, this drug may be used for related disorders, including NAFLD. To perform the characterization of OLHHA, the present study is focused on the toxicity and effects of treatment for 15 days with OLHHA on the fatty liver of obese Zucker rats. Our results show that: (1) OLHHA has a safe pharmacological profile and reduces both plasma triglyceride levels and hepatic lipid accumulation in the obese rats; (2) OLHHA induces changes in the mRNA and protein expression of enzymes related to lipid metabolism in the liver; and (3) OLHHA has an anti-apoptotic effect in the liver. These results suggest that OLHHA may be a novel drug with a potential therapeutic application for the treatment of NAFLD.

## RESULTS

### Effects of OLHHA on cytochrome P450 activity

We did not find any interference on fluorescence and quenching by OLHHA. The IC_50_ value of this drug was higher than the values exhibited by the control inhibitors (supplementary material Table S1). These results suggest that OLHHA might act as a moderate/weak CYP inhibitor. Consequently, OLHHA is unlikely to induce drug-drug interactions that might produce adverse reactions or toxicity.

### Effects of OLHHA on hERG

The IC_50_ value (>150 μM) of this drug was higher than the values of the standard control inhibitors (IC_50_ value: amiodarone, 1.7 μM; bepridil, 2.2 μM; haloperidol, 1.9 μM; terfenadine, 1.0 μM). Thus, we did not observe any inhibitory activity of OLHHA on hERG, which suggests that this drug displays a safety profile.

### Effects of OLHHA on body weight and food intake

**Fig. 1.**
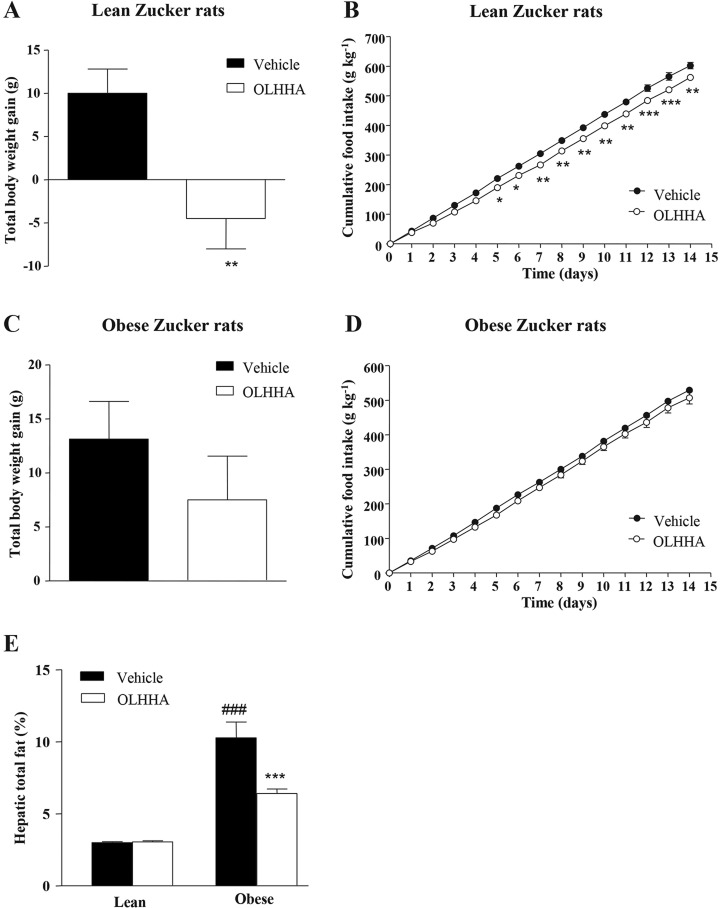
**Effects of OLHHA on body weight, food intake and hepatic fat in Zucker rats.** Total body weight gain (A,C), cumulative food intake (B,D) and hepatic total fat (E) were evaluated in the lean and obese rats after a 15-day exposure to vehicle or OLHHA (5 mg kg^−1^, daily, i.p.). The bars and points are the means±s.e.m. (*n*=8 rats per group). The data were analyzed using two-way ANOVA (treatment and time/genotype) and a *post hoc* test for multiple comparisons. *P<0.05, **P<0.01 and ***P<0.001 denote significant differences compared with the vehicle-treated rats. ^###^*P<*0.001 denotes significant difference compared with the vehicle-treated lean rats.

In the lean rats, OLHHA significantly reduced the body weight gain (*t*_14_=3.22, **P=0.006; Fig. 1A). Regarding food intake, the results of the two-way ANOVA showed a main effect of treatment (*F*_14,210_=131.5, *P<*0.001) and a significant interaction between treatment and time (*F*_1,210_=1.77, *P=*0.044; Fig. 1B). In fact, we observed that treatment with OLHHA reduced the food intake, an effect that was significant from the fifth day of treatment (*P<0.05, fifth and sixth day; **P<0.01, seventh to 11th day and 15th day; ***P<0.001, 13th and 14th day).

In the obese rats, treatment with OLHHA produced no effect on body weight gain (Fig. 1C), and although the two-way ANOVA showed a significant main effect of treatment on cumulative food intake (*F*_14,210_=29.45, *P<*0.001), the *post hoc* test comparisons revealed no significant effects when compared with the vehicle-treated rats (Fig. 1D).

### Effects of OLHHA and genotype on biochemical parameters in the plasma

To characterize the metabolic state of OLHHA-treated rats, we evaluated the levels of certain adipokines, indices of toxicity and relevant parameters related to glucose and lipid metabolism in the plasma (Table 1).

**Table 1.**
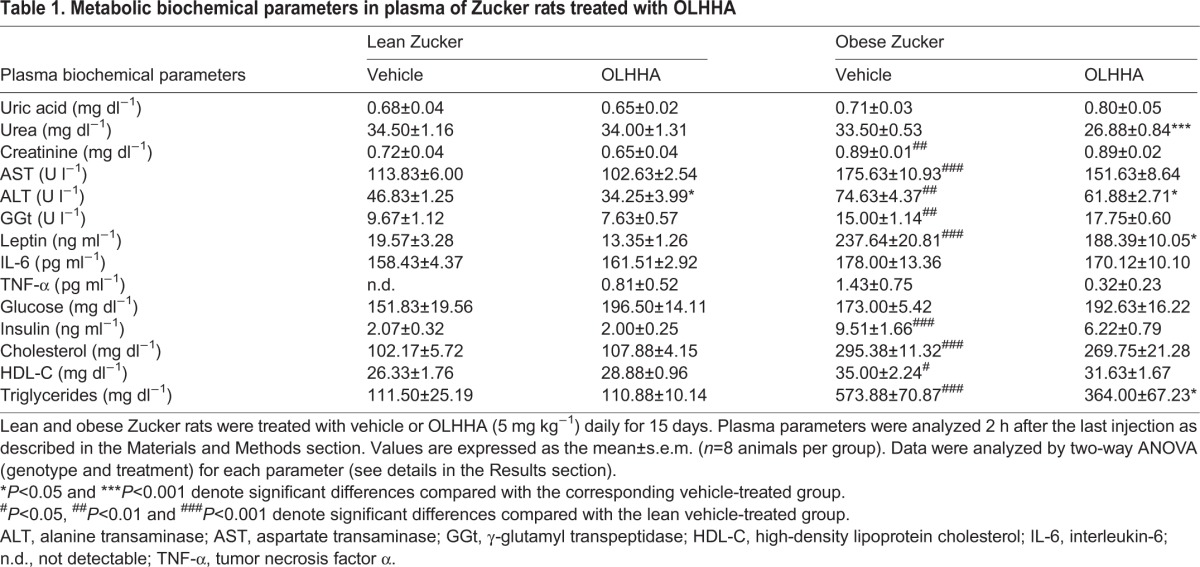
**Metabolic biochemical parameters in plasma of Zucker rats treated with OLHHA**

#### Parameters related to toxicity

The results of the two-way ANOVA showed that both genotype and treatment had significant effects on biochemical parameters related to toxicity in the plasma. Genotype produced significant effects on all of the parameters evaluated [uric acid: *F*_1,26_=5.96, *P=*0.022; urea: *F*_1,26_=16.51, *P<*0.001; creatinine: *F*_1,26_=48.72, *P<*0.001; aspartate transaminase (AST): *F*_1,26_=47.86, *P<*0.001; alanine transaminase (ALT): *F*_1,26_=60.96, *P<*0.001; γ-glutamyl transpeptidase (GGt): *F*_1,26_=77.02, *P<*0.001], and the paired comparisons revealed that the obese rats had increased levels of creatinine (^##^*P<*0.01), AST (^###^*P<*0.001), ALT (^##^*P<*0.01) and GGt (^##^*P<*0.01) compared with the vehicle-treated lean rats. In addition to genotype, treatment had a main effect on urea (*F*_1,26_=12.69, *P=*0.001), AST (*F*_1,26_=4.83, *P=*0.037) and ALT concentrations (*F*_1,26_=12.73, *P=*0.001). Thus, OLHHA reduced urea concentrations significantly (***P<0.001) in the obese rats and ALT concentrations in both lean and obese rats (*P<0.05) compared with their respective vehicle groups. Additionally, a significant interaction between genotype and treatment was observed (*F*_1,26_=9.38, *P=*0.005) and GGt (*F*_1,26_=7.40, *P=*0.012).

#### Adipokines

Both genotype (*F*_1,26_=261.7, *P*<0.001) and treatment (*F*_1,23_=5.21, *P*=0.032) had significant effects on leptin levels. Although the levels of leptin were increased in the plasma of the obese rats (^###^*P*<0.001) compared with the vehicle-treated lean rats, OLHHA reduced this increase in the obese rats (*P<0.05). The other cytokines [i.e. interleukin-6 and tumor necrosis factor α (TNF-α)] were not affected by genotype or treatment.

#### Parameters related to glucose metabolism

The ANOVA showed that treatment with OLHHA induced a significant effect on glucose levels (*F*_1,26_=5.09, *P=*0.033), but paired comparisons did not show significant differences among subgroups. With regard to insulin levels, genotype had a main effect (*F*_1,26_=24.26, *P<*0.001), increasing the insulin concentrations significantly in the obese rats (^###^*P<*0.001) compared with the vehicle-treated lean rats. Although OLHHA caused an apparent decrease in the insulin concentrations of the obese rats, it was not significant.

#### Parameters related to lipid metabolism

With regard to the parameters related to cholesterol, genotype had a significant main effect on total cholesterol (*F*_1,26_=175.5, *P<*0.001) and high-density lipoprotein cholesterol concentrations (*F*_1,26_=10.76, *P=*0.003). Indeed, the obese rats displayed higher levels of both cholesterol (^###^*P<*0.001) and high-density lipoprotein cholesterol concentrations (^#^*P<*0.05) than lean rats. However, there was no effect of treatment on these molecules. Finally, the plasma concentrations of triglycerides were significantly affected by genotype (*F*_1,28_=49.80, *P<*0.001) and treatment (*F*_1,28_=4.31, *P=*0.047). In addition, an interaction between both factors was also detected (*F*_1,28_=4.26, *P=*0.048). The paired comparisons showed that plasma triglyceride concentrations were increased in the obese rats (^###^*P<*0.001) relative to the vehicle-treated lean rats. However, treatment with OLHHA reduced significantly the increased triglyceride concentrations (*P<0.05) in the obese rats.

### Effects of OLHHA and genotype on fatty liver

#### Total fat content in the liver

Similar to plasma triglyceride concentrations, the total fat in the liver was also affected by genotype (*F*_1,28_=85.16, *P<*0.001) and treatment (*F*_1,28_=11.01, *P=*0.003), and there was an interaction between genotype and treatment (*F*_1,28_=11.53, *P=*0.002). As shown Fig. 1E, the obese rats displayed a significant increase in the total hepatic fat content compared with lean animals (^###^*P<*0.001). However, the treatment of the obese rats with OLHHA reduced the fat content significantly (***P<0.001).

#### Histological analysis

**Fig. 2.**
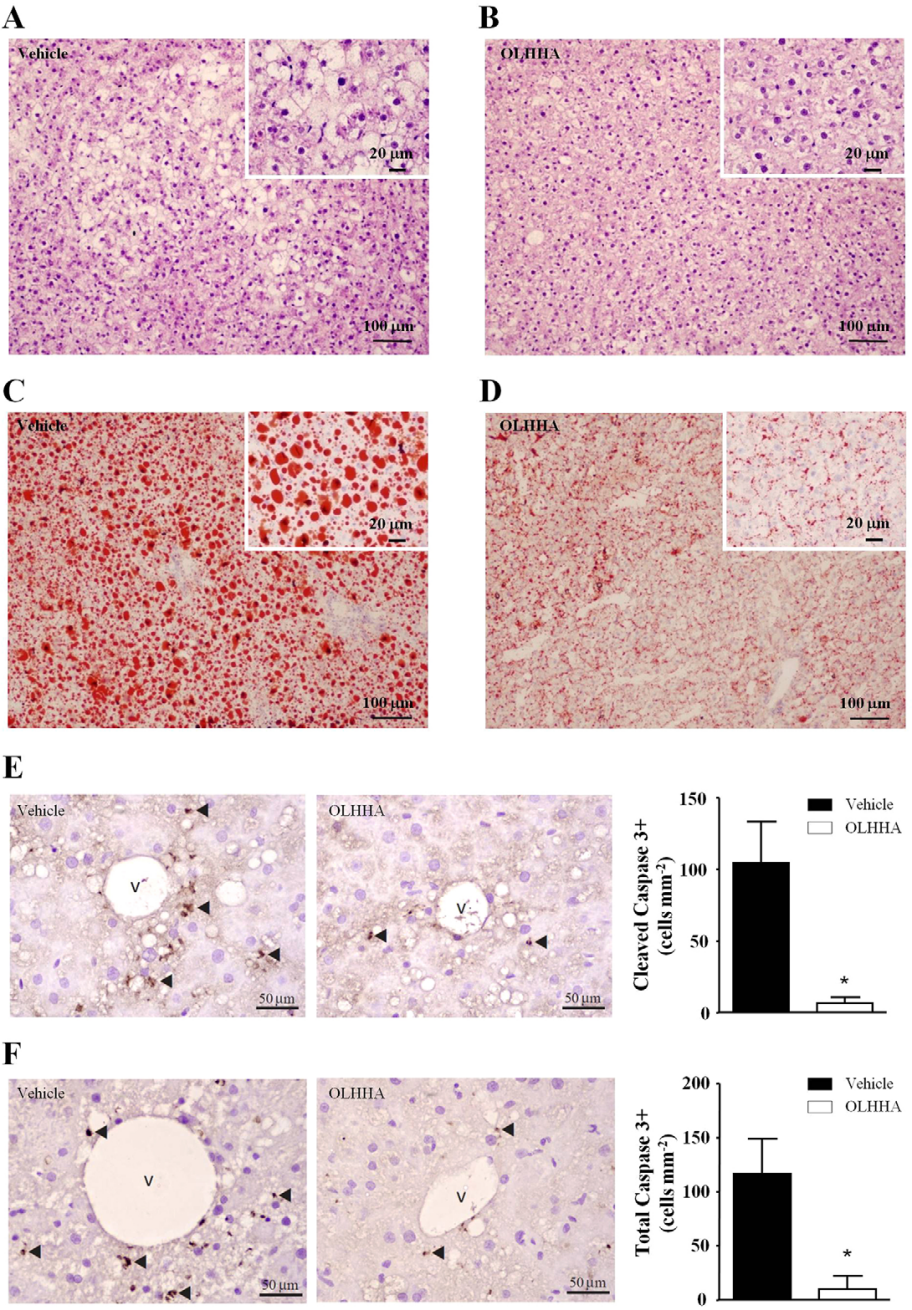
**Effects of OLHHA on the fatty liver in obese Zucker rats.** Representative images after the histological and immunohistochemical analysis of liver sections. The histological analysis of liver sections was performed using Hematoxylin and Eosin (A,B) and Oil Red O stain (C,D) to examine the effects of OLHHA on the size, number and appearance of hepatocytes and fat vacuoles in the obese rats. The immunohistochemical analysis of liver sections was performed to detect cleaved caspase-3 (E) and total caspase-3 (F) to examine the apoptotic state after a 15-day exposure to vehicle or OLHHA (5 mg kg^−1^, daily, i.p.). The scales are indicated in each image. The arrows indicate positive cells (brown stain). The bars indicate the means±s.e.m. (*n*=3 determinations in duplicate per group). The data were analyzed using Student's *t*-test. *P<0.05 denotes significant differences compared with the vehicle-treated rats.

Given that an increase in the hepatic fat accumulation was observed in the obese rats, liver sections were evaluated using histological staining with hematoxylin and eosin (Fig. 2A,B) and Oil Red O (Fig. 2C,D). Consistent with the biochemical analysis, the histological evaluation showed that treatment with OLHHA improved the fatty liver in the obese rats, with a decrease in the size of fat vacuoles but no change in the number of hepatocytes.

#### Immunohistochemical analysis of caspase-3

Complementary to the histological characterization of the liver, we assessed apoptotic activity using immunohistochemistry of cleaved (Fig. 2E) and total caspase-3 expression (Fig. 2F). Quantification of the number of cleaved and total caspase-3-positive cells in the liver sections revealed a significant decrease in the obese rats treated with OLHHA (cleaved caspase-3: *t*_4_=3.36, *P=0.028; and total caspase-3: *t*_4_=3.06, *P=0.038) relative to the vehicle-treated obese rats. It should be clear that the vehicle-treated obese rats displayed an increased caspase-3 activity compared with the lean rats (data not shown).

Therefore, the repeated treatment of obese rats with OLHHA induced a decrease in the fat content of the fatty liver by decreasing the size of fat vacuoles and by an anti-apoptotic activity.

### Effects of OLHHA and genotype on CB_1_ and PPAR-α expression in the liver

Given that OLHHA has been reported to have dual activity as a ligand for CB_1_ and PPAR-α, we evaluated the expression of the mRNA and the protein of both receptors in liver sections of lean and obese rats. The mRNA expression of *CB*_*1*_ and *PPAR-α* was affected by treatment in both genotypes. However, the protein expression of these receptors was found to be affected in different ways by treatment.

**Fig. 3.**
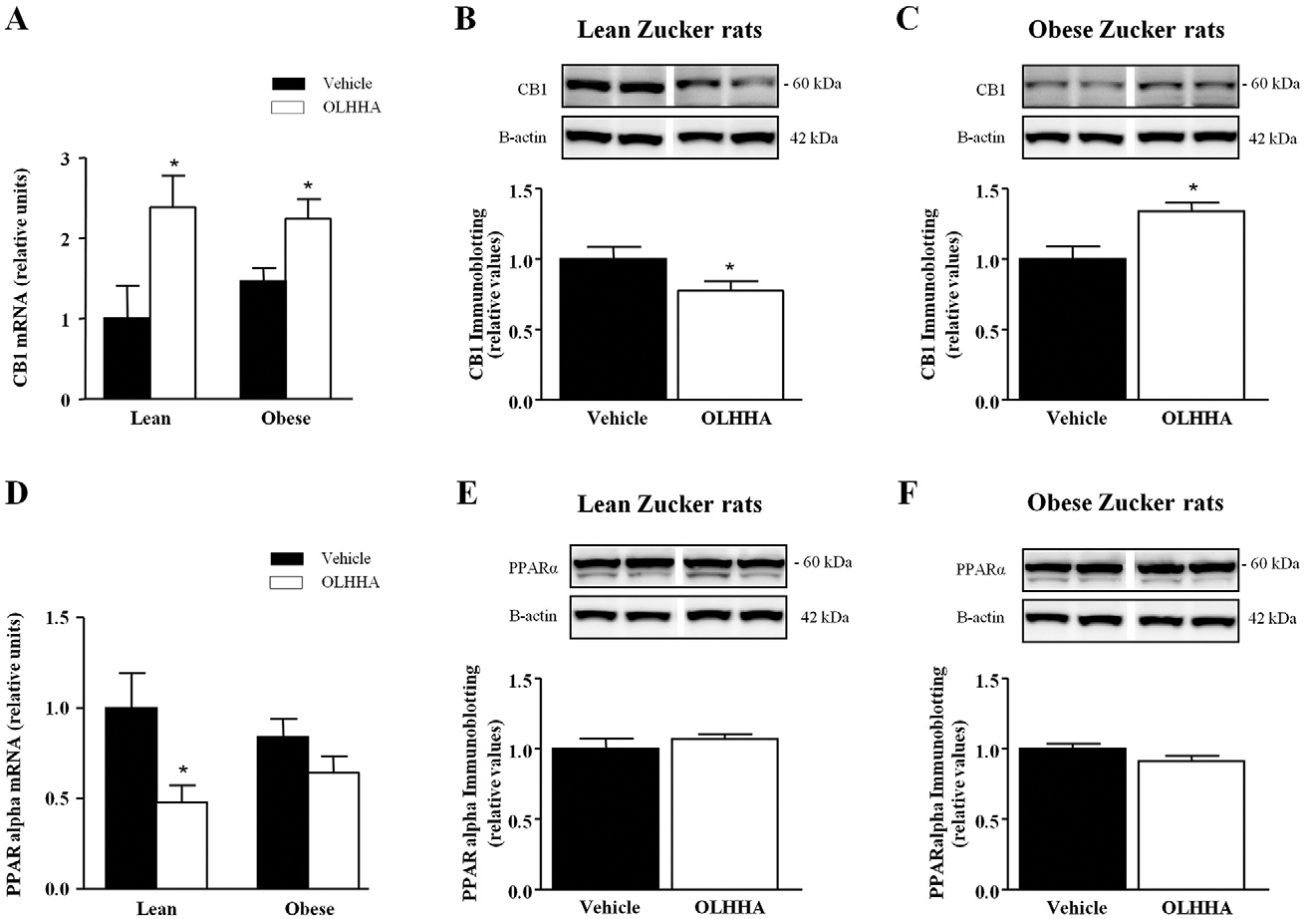
**Effects of OLHHA on the mRNA and protein expression of CB_1_ and PPAR-α in the liver of Zucker rats.** Relative mRNA levels and representative immunoblots with relative protein expression of CB_1_ and PPAR-α were determined in the lean and obese rats after a 15-day exposure to vehicle or OLHHA (5 mg kg^−1^, daily, i.p.). The mRNA expression of *CB*_*1*_ (A); and the immunoblots of CB_1_ and β-actin in both lean (B) and obese rats (C). The mRNA expression of *PPAR-α* (D); and the immunoblots of PPAR-α and β-actin in both lean (E) and obese rats (F). The bars are the means±s.e.m. (*n*=6-8 determinations per group). The data were analyzed using two-way ANOVA (treatment and genotype) and a *post hoc* test for multiple comparisons or Student's *t*-test. *P<0.05 denotes significant differences compared with the vehicle-treated rats.

As shown in Fig. 3A, gene expression of *CB*_*1*_ was significantly affected by treatment (*F*_1,23_=14.58, *P=*0.001), and OLHHA treatment increased the mRNA levels of *CB*_*1*_ in both genotypes (*P<0.05). Quantification of protein indicated a differential effect of treatment in the lean rats (Fig. 3B) compared with the obese rats (Fig. 3C). OLHHA induced a significant decrease in the relative expression of CB_1_ in the lean rats (*t*_10_=2.36, *P=0.038), whereas a significant increase was observed in the obese rats (*t*_10_=3.12, *P=0.011) relative to the respective vehicle-treated rats.

Similar to CB_1_, genotype did not affect the mRNA expression of *PPAR-α* (Fig. 3D), but treatment had a significant main effect (*F*_1,23_=9.14, *P=*0.006). The *post hoc* test comparisons revealed a significant inhibition of the expression in this nuclear receptor in the lean rats (*P<0.05), which contrasts with that observed in the mRNA levels of *CB*_*1*_. In this case, we did not find differences in the immunoblots for PPAR-α between OLHHA- and vehicle-treated rats in both genotypes (Fig. 3E,F).

#### Effects of OLHHA and genotype on the mRNA expression of the endocannabinoid system in the liver

Given that expression of CB_1_ was significantly affected and that activation of CB_1_ is related to obesity-associated hepatic steatosis, we analyzed the mRNA levels of enzymes involved in the biosynthesis of endocannabinoids [diacylglicerol lipases (DGLα and DGLβ) and *N*-acyl phosphatidylethanolamine phospholipase D (NAPE-PLD)], degradative enzymes [fatty acid amide hydrolase (FAAH) and monoacylglycerol lipase (MGL)] and the cannabinoid type 2 receptor (CB_2_) (supplementary material Fig. S1).

Focusing on the effects of OLHHA, the two-way ANOVA revealed a significant main effect of treatment on the expression of *DGLα*, *DGLβ* and *MAGL* (respectively: *F*_1,23_=5.51, *P*=0.028; *F*_1,23_=5.13, *P*=0.034; and *F*_1,23_=7.751, *P*=0.011). The paired comparisons detected a significant increase in the expression of *DGLα* in the OLHHA-treated lean rats (*P<0.05) compared with the vehicle group. In contrast, a significant decrease in the mRNA expression of *MGL* was observed in the OLHHA-treated obese rats (*P<0.05). Finally, although the analysis did not reveal any significant main effect of treatment in the mRNA expression of *CB*_*2*_, a significant increase was observed in the OLHHA-treated lean rats (*P<0.05) relative to the vehicle-treated rats.

### Effects of OLHHA and genotype on the mRNA expression of lipid metabolism in the liver

We selected certain enzymes implicated in the pathways of biosynthesis of fatty acids [fatty acid synthase (FAS), acetyl-CoA carboxylase (ACC) and stearoyl-CoA desaturase 1 (SCD-1)], biosynthesis of cholesterol [3-hydroxy-3-methyl-glutaryl-CoA reductase (HMGCR)], lipolysis [acyl-CoA oxidase (ACOX), carnitine palmitoyl transferase 1 (CPT1) and lipoprotein lipase (LPL)] or transport of fatty acids [liver-type fatty acid-binding protein (L-FABP)].

#### Biosynthesis of fatty acids

**Fig. 4.**
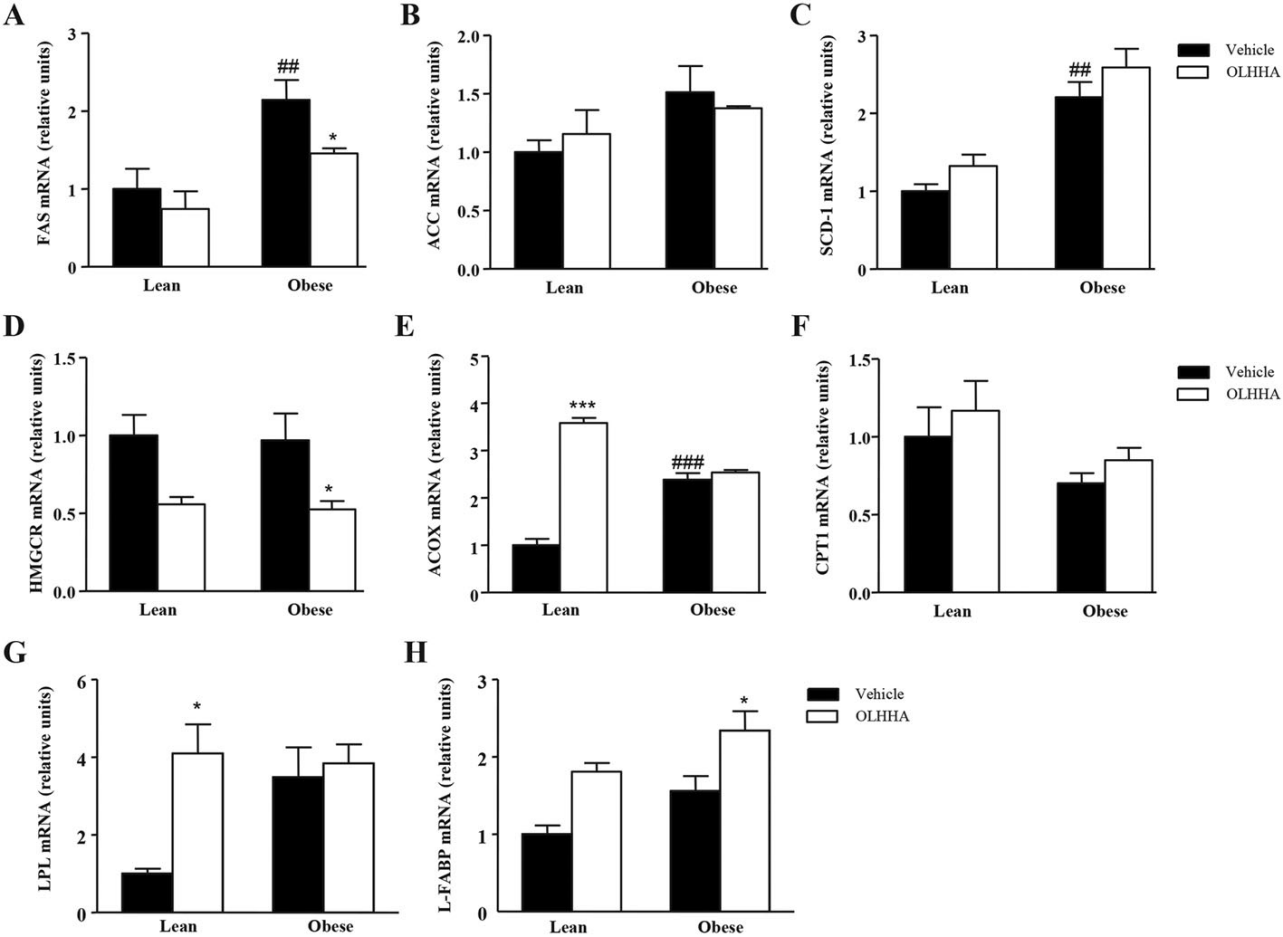
**Effects of OLHHA on the mRNA of enzymes related to lipid metabolism in the liver of Zucker rats.** Relative mRNA levels of *FAS* (A), *ACC* (B), *SCD-1* (C), *HMGCR* (D), *ACOX* (E), *CPT1* (F), *LPL* (H) and *L-FABP* (I) were determined in the lean and obese rats after a 15-day exposure to vehicle or OLHHA (5 mg kg^−1^, daily, i.p.). The bars are the means±s.e.m. (*n*=6-8 determinations per group). The data were analyzed using two-way ANOVA (treatment and genotype) and a *post hoc* test for multiple comparisons. ^##^*P<*0.01 and ^###^*P<*0.001 denote significant differences compared with the vehicle-treated lean rats; *P<0.05 and ***P<0.001 denote significant differences compared with the corresponding vehicle-treated group.

The data revealed that the mRNA expression of *FAS* was affected by genotype (*F*_1,23_=16.51, *P=*0.001) and treatment (*F*_1,23_=4.45, *P=*0.047; Fig. 4A). Thus, *FAS* expression was significantly increased in the obese rats (^##^*P<*0.01) compared with the lean rats, but this increase in *FAS* was prevented in the OLHHA-treated obese rats (*P<0.05). In contrast, the mRNA expression of *ACC* was not affected by either genotype or treatment (Fig. 4B). Regarding *SCD-1* expression, we observed only a main effect of genotype (*F*_1,23_=30.48, *P<*0.001), and the expression of SCD-1 was significantly increased in the obese rats (^##^*P<*0.01) relative to the lean rats (Fig. 4C).

#### Biosynthesis of cholesterol

The analysis of the mRNA expression of *HMGCR* showed a primary effect of treatment (*F*_1,23_=10.04, *P=*0.004), and OLHHA reduced the expression of *HMGCR*, resulting in a significant difference in the obese rats (*P<0.05; Fig. 4D).

#### Lipolysis

Treatment had a significant effect (*F*_1,23_=120.6, *P<*0.001) on *ACOX* expression, and there was a significant interaction between genotype and treatment (*F*_1,23_=95.42, *P<*0.001), but not a main effect of genotype (Fig. 4E). However, the *post hoc* test comparisons showed that the expression of *ACOX* was significantly increased in the obese rats (^###^*P<*0.001) in comparison with the vehicle-treated lean rats. Moreover, treatment with OLHHA increased the mRNA levels of *ACOX* significantly in the lean rats (***P<0.001), with no changes in the obese group. As shown in Fig. 4F, the *CPT1* expression was only affected by treatment (*F*_1,23_=6.75, *P=*0.016), but the decrease observed in the obese rats was not significant after the paired comparisons. The mRNA expression of *LPL* was significantly affected by treatment (*F*_1,23_=5.65, *P=*0.027; Fig. 4G), but this effect was only detected in the lean rats, with a significant increase in the OLHHA-treated lean rats (*P<0.05).

#### Transport fatty acids

*L-FABP* was also analyzed (Fig. 4H), and the results indicated significant effects of genotype (*F*_1,23_=5.87, *P=*0.024) and treatment (*F*_1,23_=12.50, *P=*0.002) on the mRNA expression. The multiple comparisons showed a significant increase in the OLHHA-treated obese rats (*P<0.05) compared with the vehicle group.

### Effects of OLHHA and genotype on transcription factors that regulate lipid metabolism

The expression of the mRNA of the following regulatory factors was analyzed by RT-qPCR: insulin-induced gene 1 and 2 (INSIG1 and INSIG2), sterol regulatory element-binding protein 1 and 2 (SREBP1 and SREBP2) and carbohydrate-responsive element-binding protein (ChREBP).

**Fig. 5.**
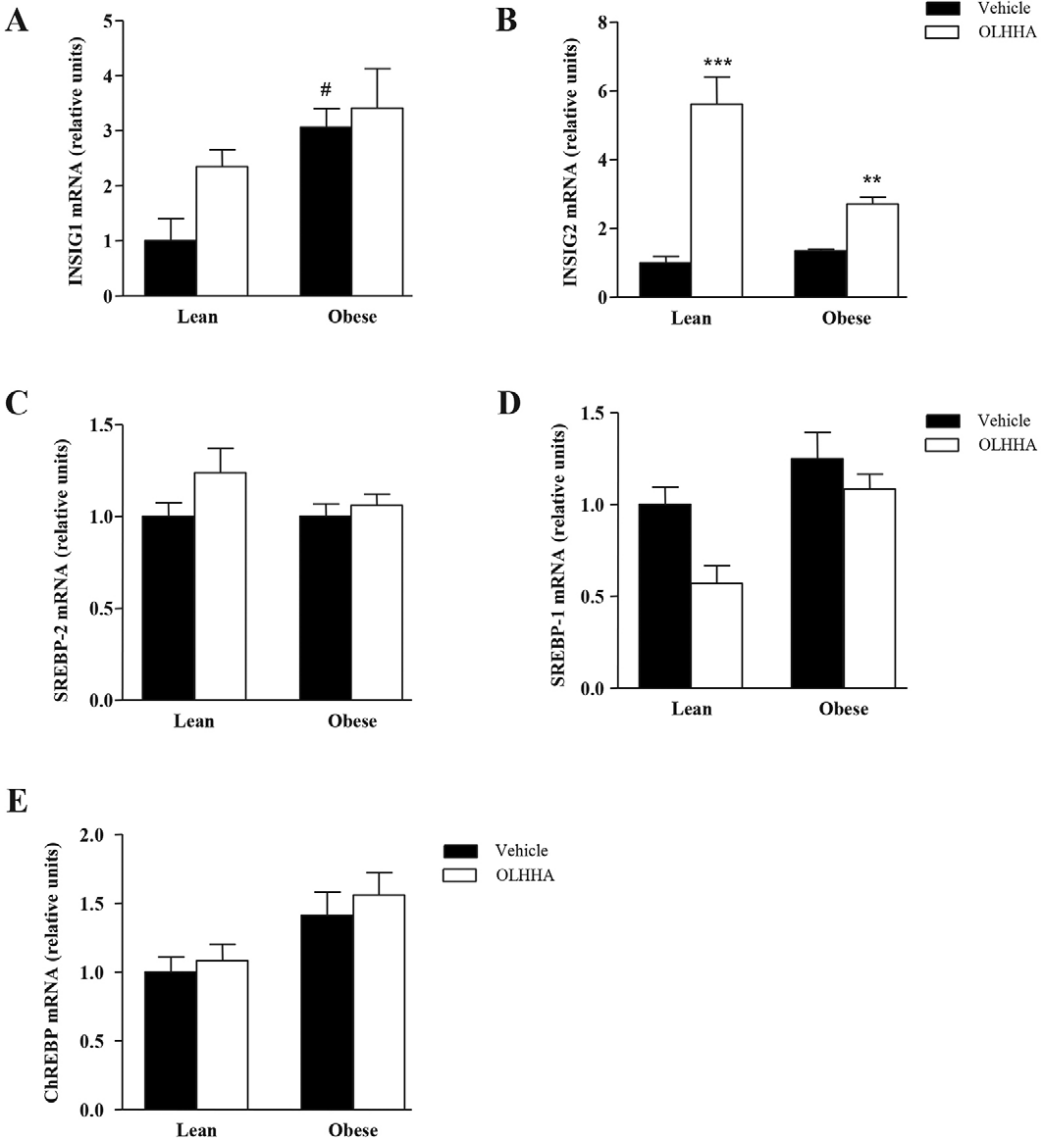
**Effects of OLHHA on the mRNA expression of regulatory factors related to lipid metabolism in the liver of Zucker rats.** Relative mRNA levels of *INSIG1* (A), *INSIG2* (B), *SREBP1* (C), *SREBP2* (D) and *ChREBP* (E) were determined in the lean and obese rats after a 15-day exposure to vehicle or OLHHA (5 mg kg^−1^, daily, i.p.). The bars are the means±s.e.m. (*n*=8 determinations per group). The data were analyzed using two-way ANOVA (treatment and genotype) and a *post hoc* test for multiple comparisons. ^#^*P<*0.05 denotes significant difference compared with the vehicle-treated lean rats; **P<0.01 and ***P<0.001 denote significant differences compared with the corresponding vehicle-treated group.

Although treatment did not affect the mRNA expression of *INSIG1* (Fig. 5A), genotype had a main effect on the regulatory factor (*F*_1,23_=7.61, *P=*0.012), resulting in a significant increase of *INSIG1* in the obese rats (^#^*P<*0.05). In contrast, *INSIG2* expression was significantly affected by both genotype (*F*_1,23_=13.73, *P=*0.001) and treatment (*F*_1,23_=75.61, *P<*0.001; Fig. 5B), with a significant interaction of both factors (*F*_1,23_=22.38, *P=*0.001). Thus, OLHHA produced a strong increase in the mRNA expression of *INSIG2* in both lean (***P<0.001) and obese rats (**P<0.01).

The ANOVA showed that the mRNA expression of *SREBP1* (Fig. 5C) was affected by both genotype (*F*_1,23_=8.77, *P=*0.007) and treatment (*F*_1,23_=5.34, *P=*0.030), with no interaction between these factors. However, the *SREBP2* expression was not affected by genotype or treatment (Fig. 5D).

Regarding *ChREBP*, the mRNA expression was affected only by genotype (*F*_1,23_=6.44, *P=*0.019; Fig. 5E), but the increase of *ChREBP* in the obese rats was not significant after the post hoc test comparisons with the lean rats.

Altogether, these data indicate that changes in the mRNA of *FAS*, *HMGCR*, *L-FAB*P and *INSIG2* are detected in the obese rats.

### Effects of OLHHA on the protein expression of enzymes and transcription factors related to lipid metabolism in the liver

**Fig. 6.**
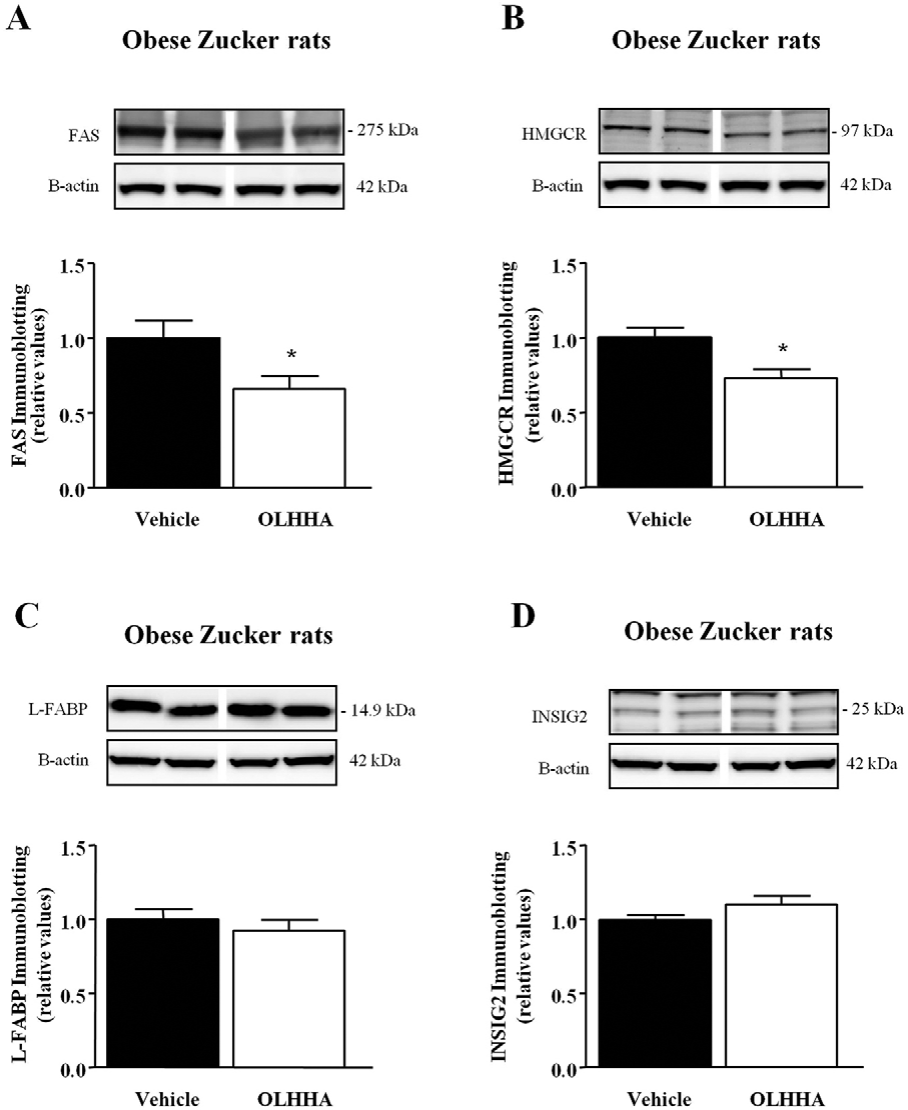
**Effect of OLHHA on the protein expression of relevant enzymes and factors related to lipid metabolism in the liver of obese Zucker rats.** Representative immunoblots and relative protein expression of FAS (A), HMGCR (B), L-FABP (C) and INSIG2 (E) were determined in the obese rats after a 15-day exposure to vehicle or OLHHA (5 mg kg^−1^, daily, i.p.). The bars are the means±s.e.m. (*n*=6 determinations per group). The data were analyzed using Student's *t*-test. *P<0.05 denotes significant differences compared with the vehicle-treated group.

To address whether the alterations of mRNA expression observed in obese rats treated with OLHHA were associated with changes in the protein expression, we analyzed the protein levels of enzymes and regulatory factors affected by this drug in the obese rats primarily. Fig. 6 illustrates representative immunoblots showing the protein expression of FAS, HMGCR, L-FABP and INSIG2 in the liver of obese rats. The obese rats treated with OLHHA displayed a significant reduction in the protein expression of FAS (*t*_11_=2.34, *P=0.042) and HMGCR (*t*_10_=2.96, *P=0.014) compared with the vehicle-treated rats (Fig. 6A,B). However, the protein levels of L-FABP and INSIG2 were not affected by treatment (Fig. 6C,D). Additionally, we performed the same protein analysis in the lean rats (supplementary material Fig. S2). The treatment of the lean rats with OLHHA only had a significant effect on the protein expression of FAS, resulting in a decrease (*t*_10_=2.59, *P=0.027).

Therefore, FAS and HMGCR are affected by OLHHA treatment, with a significant decrease in the mRNA and protein expression in the liver of obese rats.

## DISCUSSION

Although repeated OLHHA treatment reduced the food intake and body weight gain more clearly in the lean rats, we found that this drug reduced the progression of NAFLD in the obese rats, with anti-steatotic and hepatoprotective activity. Three relevant findings can be highlighted from the present study regarding the obesity-associated fatty liver. First, OLHHA treatment is a pharmacologically safe drug that ameliorates the fatty liver disease by decreasing the hepatic lipid content and circulating triglycerides and improving the profile of toxicity parameters in the plasma (e.g. transaminases ALT and AST). Second, the anti-steatotic and hepatoprotective effects of OLHHA are confirmed by a decrease in the size of hepatic fat vacuoles and anti-apoptotic activity. Third, OLHHA treatment is associated with an increase in the expression of CB_1_ and a downregulation in the expression of the lipogenic enzymes in the liver of obese rats (FAS and HMGCR, primarily).

Previously, we have reported that acute administration of OLHHA significantly decreases food intake in food-deprived Wistar rats (Almeida et al., 2010). In the present study, we performed a repeated treatment of this dihydroxyphenyl derivative in a genetic model of obesity and NAFLD, followed by initial assays related to cardio- and hepatotoxicity. Thus, we showed that OLHHA displayed a safe pharmacological profile because it did not interact with the hERG cardiac potassium channels and it had no or moderate effects on the activity of different isoforms of hepatic cytochrome P450.

A 15-day treatment with an effective dose of OLHHA produced a significant reduction in both food intake and body weight in the lean Zucker rats, whereas it had no such effect in the obese animals. The body weight reduction in the OLHHA-treated lean rats was associated with the inhibitory effect on feeding behavior, because we observed that the pair-fed rats displayed a similar reduction in body weight compared with those receiving OLHHA (data not shown). In contrast, OLHHA failed to inhibit body weight gain in the obese Zucker rats, because the reduction of food intake was not as evident as in the lean rats. Although plasma leptin was observed to be improved after OLHHA treatment, the lack of effects on body weight may be related to the malfunction of leptin signaling in the obese Zucker rat. However, we observed an anti-steatotic and hepatoprotective role of OLHHA in the obese rats with fatty liver or NAFLD. We found that the repeated treatment with OLHHA significantly decreased the fat depots in the fatty liver of obese rats as well as lowering the triglyceride concentrations in plasma.

Regarding the analysis of biochemical parameters, the data revealed strong differences in transaminases, cytokines and lipids in the plasma of both rat genotypes treated with OLHHA. The estimation of the plasma transaminase concentrations is a reliable marker of hepatocellular damage, and these concentrations were significantly higher in the obese than in the lean rats. However, OLHHA treatment ameliorated the hepatic damage as indicated by lower concentrations of transaminases (i.e. ALT). Similar effects have been previously described for OEA and rimonabant in obese Zucker rats (Gary-Bobo et al., 2007; Serrano et al., 2008a). TNF-α is a pro-inflammatory cytokine involved in the development of obesity-associated fatty liver, and its concentration is increased in genetically obese rodents (Gary-Bobo et al., 2007; Xu et al., 2002). Consistently, we also found that plasma concentrations of TNF-α were higher in the obese than the lean rats. However, OLHHA treatment induced a non-significant decrease in plasma TNF-α, which has been previously described in obese Zucker rats treated with rimonabant (Gary-Bobo et al., 2007).

These hepatoprotective effects were associated with a clear histological improvement in liver steatosis, which was primarily reflected in a decrease in the size of hepatic fat vacuoles but not in the number of cells. The anti-steatotic role of OLHHA was accompanied by anti-apoptotic effects in the liver. We observed that the number of cells expressing cleaved and total caspase-3 was significantly reduced in the liver of obese rats treated with OLHHA, which is consistent with previous studies reporting that the development and progression of NAFLD is associated with activation of apoptosis pathways (Feldstein and Gores, 2005; Guicciardi and Gores, 2005). Therefore, the reduction of lipid content and cells immunoreactive for caspase-3 might contribute to the hepatoprotective effect of OLHHA.

Similar effects have been previously reported with natural and synthetic ligands for PPAR-α, such as OEA (Fu et al., 2005; Serrano et al., 2008a) and the fibrates. However, although the fibrates constitute the main clinical approach to treat hypertriglyceridemia, the use of fibrates in NAFLD remains controversial. Whereas some studies suggest a protective role of fenofibrate in hepatic steatosis, others have reported no beneficial effects (for review, see Kostapanos et al., 2013). These opposite effects may be explained by the use of different models of NAFLD, such as dietary manipulations, genetic obesity or hereditary fatty liver. Because OLHHA has a wider pharmacological spectrum than the fibrates, the reduction of hepatic fat content observed in the present study might not be mediated exclusively by PPAR-α but derived from interaction with other targets. In fact, we have detected no substantial changes in both mRNA and protein expression of this nuclear receptor in the obese rats. Nevertheless, we propose that the effects of OLHHA are derived from an interaction between CB_1_ and PPAR-α. This is supported by previous experimental observations that the treatment of obese Zucker rats with either CB_1_ antagonist rimonabant (Gary-Bobo et al., 2007) or the combined administration of rimonabant with OEA (Serrano et al., 2008a) reduced fat depots in the liver and circulating triglycerides. The present results show an increase in CB_1_ in Zucker rats treated with OLHHA, supporting the existence of interactions between OLHHA and this cannabinoid receptor. This upregulation was indeed observed in both mRNA and protein expression in the obese rats, which has been suggested to be associated with an increase in the efficacy of CB_1_ antagonists (Kunos et al., 2008; Miller and Devi, 2011; Vickers et al., 2003). These data support the suggestion that the biological activity of OLHHA is similar to that of rimonabant. Regarding PPAR-α, we found that OLHHA treatment induced a reduction of mRNA expression, and this is a typical feature after chronic treatment with an agonist, which confirms the existence of affinity of OLHHA for these receptors. However, we detected no changes in the protein expression using immunoblot.

To explore the molecular mechanisms underlying the anti-steatotic role of the OLHHA, we investigated the expression of key enzymes that are involved in lipid metabolism and energy homeostasis in the liver. We observed that the mRNA expression of lipogenesis-related enzymes was higher in the obese rats than in the lean control animals. However, a repeated treatment with OLHHA decreased the expression of mRNA and protein of both FAS and HMGCR, enzymes involved in the biosynthesis of fatty acids and cholesterol, respectively. Regarding other enzymes, mRNA expression of *INSIG2* and *L-FABP* was also modified in the obese rats treated with OLHHA, but protein expression using immunoblot analysis was not affected. INSIG2 is a negative regulator of the cholesterol biosynthesis that binds to HMGCR, which leads to its ubiquitylation and further degradation (Dong and Tang, 2010). In agreement, the present results showed that the reduction in the expression of HMGCR was associated with *INSIG2* upregulation. In contrast, OLHHA enhanced mRNA expression of L-FABP, a protein involved in the uptake and intracellular transport of fatty acids. The gene expression of *L-FABP* is regulated by PPAR-α, and its expression has been reported to be upregulated by fibrates (Brandes et al., 1990).

Together, these data suggest that the reduction in the hepatic and circulating triglycerides observed after OLHHA treatment may be associated with the downregulation of the expression of lipogenesis-related enzymes in the fatty liver of obese rats. Similar to OLHHA, it has been well documented that OEA reduces the serum triglyceride concentrations as well as the lipid content in the liver of lean and obese rat. However, although OEA exerts these effects through a mechanism that involves PPAR-α, stimulating lipolysis and fatty acid oxidation (Fu et al., 2005; Guzman et al., 2004), OLHHA affects the lipid biosynthesis through a mechanism that differs from those described for OEA because it involves cannabinoid signaling.

As commented previously, the CB_1_ might be also involved in the mechanism of action of OLHHA. In this regard, the activation of hepatic CB_1_ is associated with an increase of *de novo* fatty acid synthesis, and this lipogenic response is abolished by CB_1_ blockade (Osei-Hyiaman et al., 2005). Given that the endocannabinoid system plays a main role in the regulation of energy homeostasis and that it has also been reported that OLHHA has affinity for the CB_1_ (Almeida et al., 2010), we evaluated the gene expression of the different components of this system in the liver of Zucker rats. Consistent with previous reports showing that the endocannabinoid system is altered in peripheral tissues in obese rats (Izzo et al., 2009, 2010), our results showed that this signaling system was dysregulated in the liver. Regarding OLHHA, we observed a strong increase in the gene expression of enzymes involved in the metabolism of 2-arachidonoylglycerol (2-AG) as well as an increase in both cannabinoid receptors.

In summary, our results show that OLHHA has an anti-steatotic activity because it is able to reverse the obesity-associated fatty liver (NAFLD) in an animal model of genetic obesity. This hepatoprotective role is associated with the downregulation of several lipogenesis-related enzymes in the liver. Although further investigations are now required to establish the mechanism and specific interactions of other pathways distinct to lipid metabolism (e.g. glucose metabolism) that contribute to its *in vivo* effects, OLHHA can be considered to represent a new class of drug with a safe pharmacological profile that may have a potential clinical application for the treatment of NAFLD.

## MATERIALS AND METHODS

### Animals and ethical statement

The experiments were performed on 8- to 9-week-old male Zucker rats [obese (*fa/fa*) and lean (*+/?*) rats] (Crl:ZUC-*Lepr^fa^*; Charles River Laboratories, Barcelona, Spain). The animals were housed individually with a 12 h-12 h light-dark cycle (lights off 20.00 h) in a room with temperature and humidity control. Water and rat chow pellets were available *ad libitum* throughout the course of the present studies.

All experimental procedures with animals were conducted in accordance with the European Community Directive 2010/63/EU and Spanish Legislation (Real Decreto 53/2013, BOE 34/11370-11421, 2013) regulating the care and use of laboratory animals. All efforts were made to minimize animal suffering, and the protocols were approved by the Ethics Committee for Animal Experiments of the Universidad de Malaga. All studies involving animals are reported in accordance with the ARRIVE guidelines for reporting experiments involving animals (Kilkenny et al*.*, 2010; McGrath et al*.*, 2010).

### Drugs

*N*-[1-(3,4-dihydroxyphenyl)propan-2-yl]oleamide (OLHHA) was synthesized as previously described (Almeida et al., 2010). OLHHA was dissolved in a vehicle containing 5% Tween 80 in saline and injected intraperitoneally (i.p.) at a dose of 5 mg per kg body weight (mg kg^−1^).

### Human cytochrome P450 (CYP) fluorometric inhibition assay

The assay was performed in collaboration with the Fundacion Medina (Centro de Excelencia en Investigacion de Medicamentos Innovadores en Andalucia, Granada, Spain). OLHHA and control inhibitors (ketoconazole, sulphafenazole and quinidine) were serially diluted by using a dilution factor of 2:1 to provide different concentrations. Dimethyl sulfoxide (0.35%) and acetonitrile (0.65%) were used as organic solvents, with a maximal concentration in the assay established as 105 μM. Both fluorescence and quenching interferences were determined for each compound in triplicate. Therefore, IC_50_ values were obtained in fluorometric CYP inhibition assays with three different CYPs: CYP3A4, CYP2C9 and CYP2D6.

### Human ether-à-go-go related gene (hERG) channel assay using a cell fluorescence functional assay

This study was performed in collaboration with the above-mentioned institution. HEK-293 (human embryonic kidney) cells expressing hERG K^+^ channels were seeded into poly-d-lysine-coated 96-well plates. The FluxOR potassium channel assay was performed as outlined in the product information sheet (available from Invitrogen, Carlsbad, CA, USA), and measured at room temperature via the FLIPR Tetra System (Molecular Devices, Sunnyvale, CA, USA). After 24 h of incubation, the plates were washed with assay buffer of the following composition (mM): 165 NaCl, 4.5 KCl, 2 CaCl_2_, 1 MgCl_2_, 10 HEPES and 10 glucose, pH 7.4. Next, loading buffer containing the FluxOR dye mix was added into each well, and the cells were incubated for 1 h at room temperature. They were washed once in assay buffer and then incubated with the same buffer containing OLHHA and control inhibitors (amiodarone, bepridil, haloperidol and terfenadin) at a dilution of 1:200, on the EP3 pipetting platform. Subsequently, compounds and OLHHA were measured in triplicate in each of three independent plates seeded with hERG-expressing HEK-293 cells, using 12-point curves (1:2 dilutions, and 150 mM as maximal concentration). Astemizol (1 mM) was added to each well as the positive control, whereas 0.5% DMSO was used as negative control. Next, plates containing both control inhibitors and OLHHA were allowed to equilibrate for 30 min at room temperature. Finally, stimulation buffer (Tl_2_SO_4_+K_2_SO_4_) was added to the plates via FLIPR Tetra System for kinetic analysis during 120 s, and IC_50_ values were obtained.

### A 15-day study of feeding behavior in Zucker rats treated with OLHHA

Animals received a daily i.p. injection of vehicle or OLHHA (5 mg kg^−1^) for 15 consecutive days. Eight rats per group were used for the experiment. Food intake (in grams per kilogram of body weight) and body weight (in grams) were measured daily.

### Sample collection

The rats were killed 2 h after the last dose. OLHHA-treated and control animals were anesthetized with sodium pentobarbital (50 mg kg^−1^, i.p.), and blood and liver samples were collected. Blood was centrifuged (2100 ***g*** for 8 min, 4°C) and the plasma kept for further analysis. Liver samples were divided into two pieces: one piece was snap-frozen in liquid N_2_ and stored at −80°C until analysis; and the other piece was fixed in 4% paraformaldehyde in 0.1 M phosphate-buffered saline (PBS) by immersion for 24 h and embedded in paraffin for histological and immunohistochemical analysis.

### Biochemical analysis in plasma

The following metabolites, metabolic hormones and adipokines were measured in plasma: uric acid, urea, creatinine, aspartate transaminase (AST), alanine transaminase (ALT), γ-glutamyl transpeptidase (GGt), leptin, interleukin-6, tumor necrosis factor α (TNF-α), glucose, insulin, cholesterol, high-density lipoprotein cholesterol and triglycerides.

The metabolites were analyzed using commercial kits according to the manufacturer's instructions in a Hitachi 737 Automatic Analyzer (Hitachi, Tokyo, Japan). The levels of leptin, IL-6 and TNF-α were measured using commercial rat enzyme-linked immunosorbent assay kits (Abcam, Cambridge, UK). The plasma levels of insulin were measured using a commercial rat insulin enzyme-linked immunosorbent assay kit (Mercodia, Uppsala, Sweden).

### Liver fat extraction

Total lipids were extracted from frozen liver samples with chloroform-methanol (2:1, v/v) according to the method of Bligh and Dyer (1959). After two centrifugation steps (2800 ***g*** for 10 min, 4°C), the lower phase, containing lipids, was extracted. N_2_ was used to dry each sample, and the liver fat content was determined by subtracting the weight of the empty tube from the weight of the tube containing the total fat extraction. The data were expressed as a percentage of tissue weight.

### Histological evaluation

Liver samples embedded in paraffin were cut by microtome (5 μm thick), mounted on d-polylysinated glass slides, deparaffinized in xylene, and stained with hematoxylin and eosin. In addition to hematoxylin and eosin stain, cryostat frozen sections were stained with Oil Red O for analysis of lipids and fat depots in the liver.

### Immunohistochemical analysis

Liver samples embedded in paraffin were cut into 5-μm-thick sections using a Microm HM325 microtome (MICROM, Walldorf, Germany) and organized on glass slides. The sections were dewaxed, washed several times with Tris-buffered saline (TBS, pH 7.8), and incubated in 3% hydrogen peroxide in TBS for 20 min in the dark at room temperature in order to inactivate endogenous peroxidase. After three washes in PBS for 5 min, antigen retrieval was achieved by incubating in sodium citrate (pH 6.0) for 20 min at 80°C. A background blocker solution containing 10% donkey serum, 0.3% Triton X-100 and 0.1% sodium azide was used to incubate the sections for 1 h, which was followed by 48 h of incubation at 4°C with the primary antibody rabbit anti-cleaved caspase-3 (Novus Biologicals, Abingdon, UK) at a dilution of 1:100. Then, the sections were washed three times with TBS, incubated in a biotinylated donkey anti-rabbit IgG (Amersham, GE Healthcare Europe, Barcelona, Spain) diluted 1:500 for 90 min, washed again in TBS, and incubated in ExtrAvidin peroxidase (Sigma, St Louis, MO, USA) diluted 1:2000 in darkness at room temperature for 1 h. After three washes in TBS in darkness, we revealed immunolabeling with 0.05% diaminobenzidine (DAB; Sigma), 0.05% nickel ammonium sulfate and 0.03% H_2_O_2_ in PBS. All steps were performed through gentle agitation at room temperature. The sections were counterstained with hematoxylin. Then, they were dehydrated in ethanol, cleared in xylene and coverslipped with Eukitt mounting medium (O. Kindler, Freiburg, Germany).

Digital high-resolution photomicrographs of the liver tissue were taken with a 40× objective in the same conditions of light and brightness/contrast using an Olympus BX41 microscope equipped with an Olympus DP70 digital camera (Olympus Iberia SAU, Barcelona, Spain). Quantification of the number of cleaved/total caspase-3-positive cells was performed using random areas (1 mm^2^) from replicates and samples (two replicates per sample, three samples per group) using the analysis software ImageJ 1.38x (National Institutes of Health, Bethesda, MA, USA).

### RNA isolation and RT-qPCR analysis

Total RNA was extracted from liver samples using Trizol Reagent (Gibco BRL Life Technologies, Baltimore, MD, USA). The total mRNA concentrations were quantified using a spectrophotometer to ensure ratios of absorbance at 260 to 280 nm of 1.8-2.0.

The reverse transcription was performed using the Transcriptor Reverse Transcriptase kit and random hexamer primers (Transcriptor RT; Roche Diagnostic, Mannheim, Germany). The quantitative real-time reverse transcription polymerase chain reaction (RT-qPCR) was performed using an ABI PRISM^®^ 7300 Real-Time PCR System (Applied Biosystems, Foster City, CA, USA) and the FAM dye label format for the TaqMan Gene Expression Assays (Applied Biosystems). The absolute values from each sample were normalized relative to the housekeeping β-actin gene (*Actb*). The relative quantification was calculated using the ΔΔ*Ct* method and normalized to the control group. Primers for the RT-qPCR (supplementary material Table S2) were obtained based on the Applied Biosystems genome database of rat mRNA references (http://bioinfo.appliedbiosystems.com/genome-database/gene-expression.html).

### Protein extraction and immunoblot analysis

The liver samples were disrupted in lysis buffer supplemented with a cocktail of protease inhibitors (cOmplete Tablets; Roche Diagnostic, Mannheim, Germany). The suspension was shaken for 2 h at 4°C and centrifuged at 20,800 ***g*** for 15 min at 4°C, recovering the soluble fraction below the fat ring. Protein concentration was determined by Bradford protein assay. The extracts of protein were diluted 1:1 in 2× sample buffer containing dithiothreitol and boiled for 5 min before submitting to SDS-PAGE. Samples (50 µg of total proteins each) were resolved in gradient SDS-PAGE gels (Bio-Rad Laboratories, Madrid, Spain) and blotted onto nitrocellulose membranes (Bio-Rad Laboratories). The membranes were blocked in TBS-T (50 mM Tris-HCl, pH 7.6; 200 mM NaCl and 0.1% Tween-20) with 2% bovine serum albumin for 1 h. Specific proteins were detected through incubation in TBS-T containing 2% bovine serum albumin for 2 h with the corresponding primary antibodies: rabbit anti-FAS (Cell Signaling Technology, Danvers, MA, USA), anti-HMGCR (Abcam), anti-PPARα (Fitzgerald, Acton, MA, USA), anti-CB_1_ (Abcam), anti-INSIG2 (Santa Cruz Biotechnology, Heidelberg, Germany), anti-LFABP (Santa Cruz Biotechnology) and anti-actin (Sigma).

After extensive washing in TBS-T, anti-rabbit horseradish-peroxidase-conjugated secondary antibody (Promega, Madison, MI, USA) was added for 1 h. The membranes were subjected to extensive washings in TBS-T, and the specific protein bands were revealed using the enhanced chemiluminescence detection system (Santa Cruz Biotechnology, Dallas, TX, USA), in accordance with the manufacturer's instructions, and the images were visualized in an Autochemi-UVP Bioimaging System. Bands were quantified using a densitometric analysis through ImageJ software (Wayne S. Rasband, ImageJ; US National Institutes of Health, Bethesda, MA, USA; http://imagej.nih.gov/ij, 1997–2012). The values were expressed in relation to β-actin.

### Statistical analysis

All the data in the graphs and tables are expressed as the means±s.e.m. The statistical analysis was performed using GraphPad Prism version 5.04 (GraphPad Software, San Diego, CA, USA). The significance of differences within and between groups was primarily evaluated using two-way analysis of variance [ANOVA; factors: treatment (vehicle/OLHHA) and genotype (obese/lean) or treatment and time] and the appropriate *post hoc* test for multiple comparisons. The analysis of two groups was performed using Student's unpaired *t*-test. A *P*-value of less than 0.05 was considered significant.

## Notes

This article is part of a subject collection on Model Systems in Drug Discovery: from Bench to Patient. See related articles in this collection at http://dmm.biologists.org/cgi/collection/drugdiscovery
